# Assessing the effect of *Bacillus rugosus* NM007 as a novel probiotic strain on growth performance, hemato-biochemical profile, immune response, digestive enzymes and intestinal histomorphology of Nile tilapia (*Oreochromis niloticus*)

**DOI:** 10.1007/s11274-025-04742-8

**Published:** 2026-02-19

**Authors:** Nermeen M. Shokrak, Basant A. Bakr, Samar Tawfik, Radi A. Mohamed, Bahaa Abdella

**Affiliations:** 1 Department of Aquaculture, Faculty of Aquatic and Fisheries Sciences, Kafrelsheikh University, Kafr El-Sheikh, 33516 Egypt; 2 Zoology Department, Faculty of Science, Alexandria University, Alexandria, 21511 Egypt

**Keywords:** Aquatic health, Sustainability, Immune response, Antioxidant activity

## Abstract

With the growing global demand for seafood, sustainable aquaculture requires enhancing fish health and feed efficiency while reducing the environmental impact. The current study evaluated the potential probiotics of *Bacillus rugosus* NM007 in farmed Nile tilapia. Three different doses of *B. rugosus* NM007 supplemented feed, namely 0.1, 0.2, and 0.3 g/kg, were fed to fish (5.13 ± 0.09 g, N = 240) in triplicate and assigned as T_1_, T_2,_ and T_3,_ and kept for 90 days. The control group (C) received only the basic commercial diet without supplementation. The treated groups showed significant improvements in growth, feed utilization, and physiological health compared to the control. The T_2_ group showed the highest growth performance, with a weight gain of 45.66 ± 1.41 per fish (*P* = 0.001) compared to the control group and superior hematological values, including hemoglobin, RBCs, and WBCs. Liver function improved across all treated groups, with the lowest ALT and AST levels recorded in T_2_, while T_3_ showed the highest total protein and albumin levels. Antioxidant activity increased notably, as SOD and CAT were elevated and MDA was reduced in all probiotic-related groups. Lysozyme activity peaked in T_2_, indicating enhanced immune activity. Digestive enzyme analyses in serum and intestines were significantly higher in all treated groups compared to the control group. Histological analysis revealed improved intestinal villi structure, muscularis thickness, and lymphocyte count, with no signs of liver inflammation or toxicity. Overall, the new *B. rugosus* NM007 demonstrated strong probiotic potential by enhancing growth, antioxidant defense, immune response, digestive enzyme activity, and tissue health in Nile tilapia, suggesting its promise as a safe and effective novel probiotic for aquaculture.

## Introduction

Over the past seven years, Egyptian aquaculture has experienced an extraordinary and swift expansion. Egypt currently leads Africa in this field, ranking third globally in tilapia production and sixth globally in total aquaculture production (Wally and Akingbe 2022). Among the fish species, the Nile tilapia (*Oreochromis niloticus*) holds the most dominant position in Egypt. This is primarily due to its extensive expansion and the cultivated areas dedicated to its cultivation, which collectively contribute to the production of approximately 65% of aquaculture in the country (Prabu et al. 2019). In addition to that, Nile tilapia is considered one of the most widely produced species in the world due to its reproductive behavior, disease tolerance, growth rate, and consumer acceptance (Prabu et al. 2019). However, many challenges face this sector on the path to its sustainable growth, including frequent disease outbreaks, environmental issues, and the ever-decreasing world population of wild fish (Abdella et al. 2017, 2023; El-Wazzan et al. 2020). Solving these challenges requires employing the proper approach, which is derived from the integration of ecological concepts. One approach being discussed is the use of probiotics (Assan et al. 2022; Abdella et al. 2023, 2024).

Probiotics are defined as living microorganisms that provide health benefits to the host and its environment (Melo-Bolívar et al. 2021). Once ingested by the fish or added to the water, they improve host health status through altering the microbiota, enhancing disease resistance, improving health parameters, optimizing growth rates, and feed conversion efficiency (Jahari et al. 2019). Numerous studies have proved that supplementing diets with probiotics improves growth in Nile tilapia (Elsabagh et al. 2018; Khunrang et al. 2021; Silva et al. 2021) and improves the immune response (Kuebutornye et al. 2020a; Kord et al. 2021). In addition to that, the use of probiotics has become increasingly important in the aquaculture industry over the last two decades as an alternative to antibiotic use (Abdella et al. 2020; El-Saadony et al. 2021).

In aquaculture, the understanding and application of probiotics have been the subject of extensive research and implementation with regard to the wellbeing and profitability of cultured organisms (Elisashvili et al. 2019; Abomughaid 2020; Khalafalla et al. 2020; Zaineldin et al. 2021; Mugwanya et al. 2022). The published reports on the use of *Lactobacillus* spp. and *Bacillus* spp. proved to be extremely effective in aquaculture (Ringø et al. 2020; Ren et al. 2021; Mugwanya et al. 2022). The above-mentioned probiotics help in increasing the production rate of fish and shellfish as well as their survival rates, as they improve intestinal health and the immune system, and minimize the occurrence of diseases (Ringø et al. 2020; Ren et al. 2021; Mugwanya et al. 2022). For example, the effect of using *Lactobacillus* species is particularly known for making the intestinal environment more suitable for the absorption of nutrients and supporting the growth of beneficial bacteria (Assan et al. 2022; Abada et al. 2022). *Bacillus* spp., on the other hand, are known for their use as antimicrobial producers that can inhibit and suppress the growth of pathogenic bacteria, helping to reduce the use of antibiotics (Esakkiraj et al. 2009; Avella et al. 2010; Reda and Selim 2015; Addo et al. 2017; Kuebutornye et al. 2020b; Ghalwash et al. 2022; Elsadek et al. 2023).

In Egypt, aquaculture, a major industry, faces significant challenges, including water quality issues, disease outbreaks, and the overuse of antibiotics (Pepi and Focardi 2021; Mehrim and Refaey 2023). Probiotics can play a critical role in addressing these challenges by improving water quality, enhancing fish immune systems, and reducing the need for antibiotics. The use of these probiotics in aquaculture has been proven to be harmless to aquatic life and the environment, and offers better opportunities for disease control and fish growth compared to the use of chemicals (Vargas-Albores et al. 2021; Amenyogbe 2023). Therefore, regardless of the existence of effective probiotics, continuous research is essential to discover new probiotic candidates for use in aquaculture. The extreme diversity of aquatic species, resulting from differences in their physiological and ecological niches, further emphasizes the need for different probiotics (Ramirez-Paredes et al. 2020; Diwan et al. 2022).

New probiotics may be more targeted, as they can address specific issues that arise in various aquaculture systems, such as temperature fluctuations, changing salinities, and the emergence of new pathogens (Puvanasundram et al. 2021; Diwan et al. 2022). *Bacillus rugosus* was first discovered in 2020 (Azra et al. 2021), and there was limited information available regarding this strain. According to Shokrak et al. (2024), *B. rugosus* NM007 showed promising potential as a probiotic strain due to its ability to produce digestive enzymes and its other beneficial features for aquaculture*.* Furthermore, the industry is under more pressure to change as a result of the rising demand for seafood, which is, as was already mentioned, causing new diseases to emerge and requiring the use of more antibiotics (Abdella et al. 2020). Finding new probiotic strains with specific applications can help improve stress tolerance, feed conversion ratio, and disease protection mechanisms in fish (FAO 2020; Azra et al. 2021). Furthermore, as our understanding of microbial ecology expands, it offers an opportunity to discover even more beneficial probiotics that can contribute to the development of ideas for enhancing growth and health in aquatic environments. Therefore, conducting trials with novel probiotics is crucial to enhance the stability of aquaculture, enabling the respective sectors to achieve long-term sustainable growth and high results.

Species-specific responses play a crucial role in determining the effectiveness of probiotics in aquaculture, as the interaction between host fish and probiotic strains can vary greatly across species (Kochetkov et al. 2024; Fachri et al. 2024; Mahmoud et al. 2025). Therefore, there is an urgent need to isolate, characterize, and evaluate new probiotic candidates that have not yet been tested in fish to enhance aquaculture sustainability and health management. Previous studies have mainly focused on common species such as *Bacillus subtilis* and *Lactobacillus plantarum* (Abumourad et al. 2013; Olmos and Paniagua-Michel 2014; Nayak 2021). Exploring new alternatives that have not been tested might open the gate to even better-performing strains that can help in making the industry more sustainable. Thus, this study aims to assess the effect of the *Bacillus rugosus* NM007 strain as a probiotic on growth performance, hemato-biochemical profile, intestinal morphometry, immune response, digestive enzymes, and antioxidant activity of Nile tilapia.

## Materials and methods

### Ethical approval

The Committee on Aquatic Animal Care and Use in Research at Kafrelsheikh University, Egypt, approved the fish experimental techniques in this work, which followed the Egyptian legislation on ethics in the use and handling of fish (Approval Number: IAACUC-KSU-02224 −2022).

### Diet formulation, fish management, and experimental design

Nile tilapia (*Oreochromis niloticus*) juveniles were purchased from a private hatchery in Kafrelsheikh Governorate, Egypt. Before the experiment, the fish were adapted to the conditions in the laboratory for two weeks. A photo period of 12 h of light and 12 h of darkness was maintained throughout the study. A total of 240 fish were distributed randomly with an average weight of 5.13 ± 0.09 g were used in this experiment. The experiment was carried out in 12 glass aquariums of the same size (80 × 40 × 35 cm) with 20 fish/aquarium in triplicate over 90 days. To control for variability between tanks, we ensured uniform water quality parameters, feeding regimes, and environmental conditions across all tanks. The fish were fed a commercial diet (Table 1) and a potential probiotic, *Bacillus rugosus* NM007, previously isolated and prepared in our laboratory (Shokrak et al. 2024), with concentrations of 1 × 10^10^ CFU/g mixed by spraying to the basal diet using sunflower oil (20 mL/kg diet) at concentrations of 0 g (C: control), 0.1 g (T_1_), 0.2 g (T_2_) and 0.3 g (T_3_) per kg of diet. After the diet was prepared, it was preserved in dark, well-closed bottles and stored in dry areas in the laboratory. The diet was prepared every week according to the estimated feeding rate. A mechanical filter was used to remove fish waste, and the remaining waste was siphoned out every day. The aquariums were aerated using air stones. Fish were fed at a rate of 4% of their total biomass, with the experimental diet administered twice daily at 8:00 a.m. and 2:00 p.m. The total fish mortality of each aquarium was recorded daily during the experiment. The water quality parameters were measured daily during the experimental period and were kept around the normal average for Nile tilapia. Dissolved oxygen (DO), PH, and temperature were measured in the center of each aquarium using a multi-parameter probe meter (HI9829-03042-HANNA® instruments, www.hannainst.com). A portable colorimeter (Martini MI 405) was used to measure the total ammonia nitrogen. The following values (mean ± SE) were recorded and maintained: temperature 27.22 ± 0.56 °C, pH 7.90 ± 0.23, dissolved oxygen 6.11 ± 0.45 mg/L, and total ammonia nitrogen 0.031 ± 0.001 mg/L.

**Table 1:** Formulation and proximate composition of the basal diet fed to Nile tilapia (on a dry matter basis)

Ingredient	%	Chemical composition	%
Fish meal (60% CP)	3.0	Dry matter	90.0
Soybean meal	36.5	Crude protein	30.0
Corn gluten	8.1	Ether extract	6.02
Yellow corn	12.2	Crude fiber	4.95
Wheat middlings	22.5	Ash	5.1
Poultry byproducts meal	4.1	Total carbohydrates	53.93
Rice bran	8.0	Available phosphorus	0.4
Soy oil + rapeseed oil	2.0	Calcium	0.99
Mono-calcium phosphate	0.6	Gross energy (MJ kg^−1^)	18.73
Common salt	0.5		
Calcium carbonate	0.5		
^1^Premix	2.0		

^1^Premix (mg kg^−1^): vitamin A (4800 IU), vitamin D3 (800 IU), vitamin E (200 mg), vitamin B1 (133 mg), vitamin B2 (580 mg), vitamin B6 (410 mg), vitamin B12 (50 mg), biotin (9330 mg), choline chloride (4000 mg), vitamin C (500 mg), inositol (330 mg), para-amino benzoic acid (9330 mg), niacin (26.60 mg), pantothenic acid (2000 mg), manganese (325 mg), iron (200 mg), copper (25 mg), iodine, cobalt (5 mg). The premixes are provided by New Hope, Singapore premix Pte Ltd

### Measured parameters

#### Fish growth performance and feed utilization efficiency

After 90 days of the experiment, the fish were collected from the aquariums, and the fish were euthanized with clove oil (Merck) 50 μL/L (Elkaradawy et al. 2022). Each fish was weighed separately to determine the initial weight of each fish at the beginning of the feeding experiment and the final body weight of each fish at the end of the experiment. The total length (L) of the collected fish was also estimated using a measuring board from the head of the fish downstream to the tail. Growth performance and feed conversion were calculated according to (Elkaradawy et al. 2021) using the following equations.

Body weight gain (BWG) = final body weight (W_1_) − initial body weight (W_0_). Specific growth rate (SGR %/day) = 100 × (lnW_1_– lnW_0_)/t, where ‘t’ is the experimental period (days). Feed intake (g/fish) = (total daily feed intake for each group/number of fish in the same group) × t. Feed conversion ratio (FCR) = feed intake (g)/BWG (g). Protein efficiency ratio (PER) = weight gain (g)/protein intake (g). Condition factor (K) = 100 × (W_1_/L^3^). The hepato-somatic index (HSI) = 100 × (liver weight/W_1_). Viscero-somatic index (VSI) = 100 × (intestine weight/W_1_). Survival rates (SR %) = (total number of fish at the end of the experiment/total number of fish at the start of the experiment) × 100.

#### Intestinal histomorphology

The liver and intestine of nine fish per treatment were meticulously dissected. After being cleaned of unwanted substances or blood using ice-cold 1.15% KCl, the samples were dried on filter paper. The samples were fixed in 10% neutral buffered formalin for 12 to 24 h. The organs were then processed and embedded in paraffin wax and placed in tissue cassettes (Bancroft and Gamble 2008).Using a microtome (Leica “RM2125 RTS”, Germany), the embedded samples were divided into sections of 5 μm. Eosin and hematoxylin were used to stain the sections (Li et al. 2014). Using a Lecia DM750 P light microscope with a camera, the stained slides were viewed and photographed.

Measurements of villus height (from tip to base), villus width at the tip, villus width at the crypt/villus junction, and muscularis layer thickness were also used to evaluate changes in intestinal histomorphometry. Two different observers manually analyzed photomicrographs using ImageJ software to perform quantitative analysis (Bankhead et al. 2017; Mohamed et al. 2021).

##### Blood sampling and serum separation

At the end of the experimental period, nine fish per treatment were randomly sampled for blood collection from the caudal vein, three fish per aquarium. Ethylenediaminetetraacetic acid (EDTA) vacuum tubes were used as anticoagulants for hematological analysis, while plain tubes without anticoagulant were used for serum collection. Blood samples that had clotted were then centrifuged at 704 × g for 15 min at 4°C. The supernatant serum was collected into microcentrifuge tubes and preserved in a −20°C freezer until further use (Ghalwash et al. 2022).

##### Hematological analysis

The red blood cells (RBCs) counts, hemoglobin (Hb) concentration, total white blood cells (WBCs) counts, and differential WBCs counts, including lymphocytes and heterophils, were determined by a blood cells counter (Exigo-Vet, Boule Medical AB Inc., Stockholm, Sweden).

##### Serum biochemical analysis

The enzyme activities of aspartate aminotransferase (AST) and alanine aminotransferase (ALT) were monitored colorimetrically at 540 nm using commercially available kits (CAT. No. AL 10 31 (45), Biodiagnostic Co. Egypt). Serum levels of total proteins, alkaline phosphatase (Alk.ph), and bilirubin were determined at 540 nm, while serum albumin at 550 nm, according to Reitman and Frankel (1957) using commercial kits (REF:310 001 Spectrum, Egyptian company for Biotechnology, Egypt and CAT. No. AB 10 10, Biodiagnostic Co. Egypt). The globulin content was determined by the difference between total protein and albumin. Total serum cholesterol levels were assayed by GPO-PAP and CHOD-PAP methods using commercial clinical kits. Serum urea and creatinine were assayed using a colorimetric technique (Heinegård and Tiderström 1973) whereas serum glucose was estimated with commercial glucose enzymatic PAP kits obtained from Bio-Mérieux, France.

##### Antioxidant capacity

Measuring antioxidants as catalase (CAT), glutathione peroxidase (GPx), malondialdehyde (MDA), and superoxide dismutase (SOD) in Nile tilapia serum is a common way to assess oxidative stress, often using commercial ELISA (Enzyme-Linked Immunosorbent Assay) kits that provide high-sensitivity and specificity. To evaluate the activities of CAT, MDA, GPx and SOD, nine fish were chosen randomly from each treatment group and assayed using ELISA Kits purchased from (Inova Biotechnology, China). These samples were read at 450 nm with a microplate ELISA reader (PR 4100 ELISA Reader, Bio-Rad, USA) (Abdel-Tawwab et al. 2018). A standard curve is plotted from the known standards, and the concentration of antioxidants in the tilapia serum is interpolated from this curve.

### Digestive enzymes activity

#### Digestive enzymes activity in serum

Digestive enzyme activities in serum were measured (9 fish from each treatment) using the diagnostic reagent kits purchased from Cusabio Biotech Co. Ltd., Wuhan, Hubei, China, based on the manufacturer’s instructions. As described by Abdel-Tawwab et al. (2018), assays of lipase and amylase activities were determined. Lipase was quantified spectrophotometrically with absorbance set at 580 nm, while the amylase activity assay was based on the 660 nm absorbance (Moss and Henderson 1999).

#### Digestive enzymes activity in intestine

Intestinal enzyme activities were determined in nine fish per treatment group. The intestines were aseptically removed and washed in the PBS buffer (pH 7.5; 1 g/10 mL), homogenized for 20 min, then centrifuged at 8000 g for 5 min. The supernatant was stored at 4 °C. The activity of proteases was determined using the Folin phenol reagent (Anson 1938). Amylase activity was determined according to Jiang (1982) and Dawood et al. (2019), through estimating the amount of reacted starch by iodine solution. Enzyme measurement of lipase was determined by the method described in (Borlongan 1990; Jin 1995) using olive oil as a substrate. All results were expressed relative to the control group.

#### Immune response

To evaluate the impact of the *Bacillus rugosus* NM007 probiotic on the fish’s immune response, the blood of nine fish from each treatment was randomly chosen for the experiment. Serum lysozyme activity was determined using the ELISA by a microplate ELISA reader at 450 nm according to the procedure described by (Demers and Bayne 1997).

#### Whole body composition analysis

At the end of the trial, five fish from each group were chosen for chemical body composition analysis. The fish were then stored in a deep freezer at −20 °C until the time of usage. Crude protein, crude fat, carbohydrates, moisture and ash content of the entire fish body were determined by (AOAC 1957) procedures.

### Statistical analysis

The data were assessed visually for normal distribution using QQ plot. Residuals were also analyzed to confirm normality. All percentage values were transformed using angular transformation for further analysis. Statistical analyses were conducted using GraphPad Prism v7.0 and SPSS (IBM SPSS Statistics v22). Results are presented as mean ± SEM. To compare different treatments, one-way ANOVA was applied, followed by Tukey’s multiple comparison test when applicable. Statistical significance was defined as *P* < *0.05*. Post-hoc power analysis was not performed, as the sample size was predetermined based on previous studies to ensure adequate statistical reliability.

## Results

### Fish growth performance and feed utilization efficiency

As shown in Table 2, supplementation with *Bacillus rugosus* NM007 significantly improved several growth parameters of Nile tilapia. The T_2_ group (0.2 g/kg feed) achieved the highest weight gain (45.66 ± 1.41 g; *P* = 0.001), specific growth rate (2.57 ± 0.03; *P* = 0.008), protein efficiency ratio (3.27 ± 0.13; *P* = 0.001), and visceral somatic index (VSI) (5.14 ± 0.28; *P* < 0.001). The same group also recorded the most efficient FCR (1.02 ± 0.01; *P* < 0.001), and condition factor (K) was also the highest in T_2_ (1.92 ± 0.03; *P* = 0.038). The survival rate was 100% across all experimental groups, with no mortality observed throughout the study period.

**Table 2:** Effect of potential probiotic *B. rugosus* NM007 on growth performance of Nile tilapia (*Oreochromis niloticus*) after 90 days experimental trial compared to the control group

Factor	C	T_1_	T_2_	T_3_	*P-value*
IBW (g)	5.13 ± 0.09	4.60 ± 0.30	4.96 ± 0.03	4.76 ± 0.14	0.233
FBW(g)	38.40 ± 0.20^b^	48.15 ± 1.67^a^	50.62 ± 1.40^a^	48.39 ± 1.72^a^	0.001
WG (g)	33.27 ± 0.28^b^	43.55 ± 1.98^a^	45.66 ± 1.41^a^	43.63 ± 1.58^a^	0.001
FI (g)	49.57 ± 0.22	49.65 ± 0.14	46.57 ± 0.23	47.99 ± 0.26	0.949
FCR	1.49 ± 0.04^a^	1.14 ± 0.01^b^	1.02 ± 0.01^c^	1.10 ± 0.01^bc^	< 0.001
SGR (%/day)	2.23 ± 0.02^b^	2.61 ± 0.11^a^	2.57 ± 0.03^a^	2.57 ± 0.01^a^	0.008
PER (%)	2.24 ± 0.01^c^	2.92 ± 0.19^b^	3.27 ± 0.13^a^	3.03 ± 0.15^b^	0.001
FL (cm)	13.26 ± 0.46^b^	13.59 ± 0.29^ab^	13.80 ± 0.17^a^	14.71 ± 0.96^ab^	0.013
K	1.66 ± 0.16^a^	1.91 ± 0.06^ab^	1.92 ± 0.03^b^	1.59 ± 0.27^ab^	0.038
HSI (%)	1.05 ± 0.10	1.32 ± 0.17	1.18 ± 0.14	1.56 ± 0.22	0.204
VSI (%)	2.16 ± 0.17^c^	3.57 ± 0.27^b^	5.14 ± 0.28^a^	3.59 ± 0.30^b^	< 0.001
Survival rate (%)	100	100	100	100	

For means in the same row, those with no common superscripts are significantly different at *P* < 0.05

Abbreviations: *IBW* Initial body weight; *FBW* Final body weight; *WG* Weight gain; *FCR* Feed conversion ratio; *FI* Feed intake; *SGR* Specific growth rate; *PER* Protein efficiency ratio; *FL* Fish length; *K* Condition factor; *HSI* Hepatosomatic index; *VSI* Viscerosomatic index

*C* Control, *T*_1_ Treatment 1, (0.1 g/kg of feed), *T*_2_ Treatment 2, (0.2 g/kg of feed), *T*_3_ Treatment 3, (0.3 g/kg of feed)

### Intestinal histomorphology and liver toxicity

The histo-morphological changes of the intestine and liver of Nile tilapia fed by the experimental diets with *Bacillus rugosus* NM007 probiotic are shown as follows:

#### Qualitative histometric analysis of intestine

**Fig. 1:**
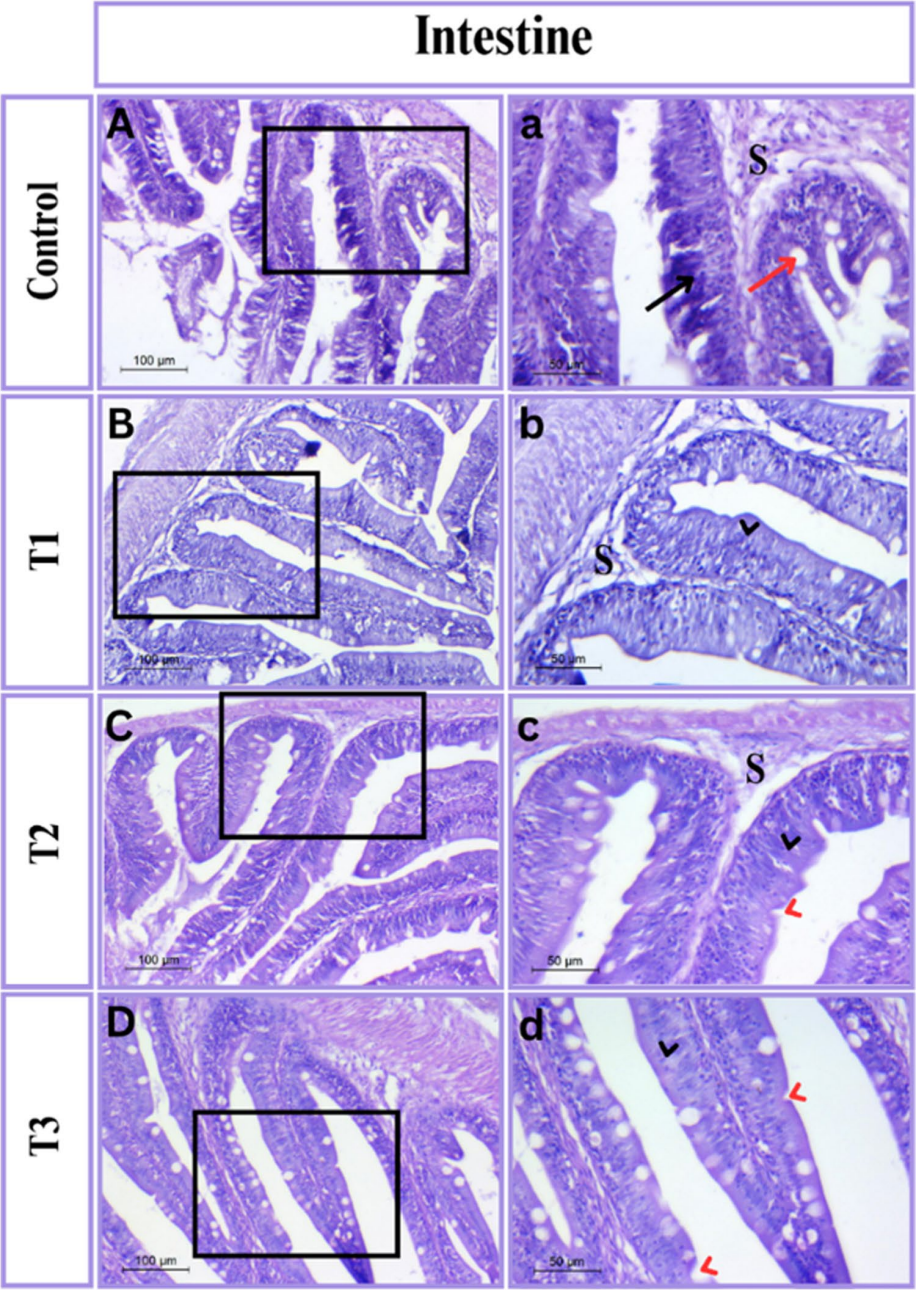
Photomicrograph of intestinal tissue in Tilapia stained with H&E stain. The right panels (magnification X 200) are higher magnification of the insets of the left panels (magnification X 100). (**A**-**a**) control group; (**B**-**b**) T_1_ group; (**C**–**c**) T_2_ group and (**D**-**d**) T_3_ group. Representative samples illustrated by some symbols: irregular columnar epithelia (black arrow); lymphocytes (red arrow); submucosa (S); normal columnar epithelia (black head arrow) and goblet cells (red head arrow)

**Fig. 2:**
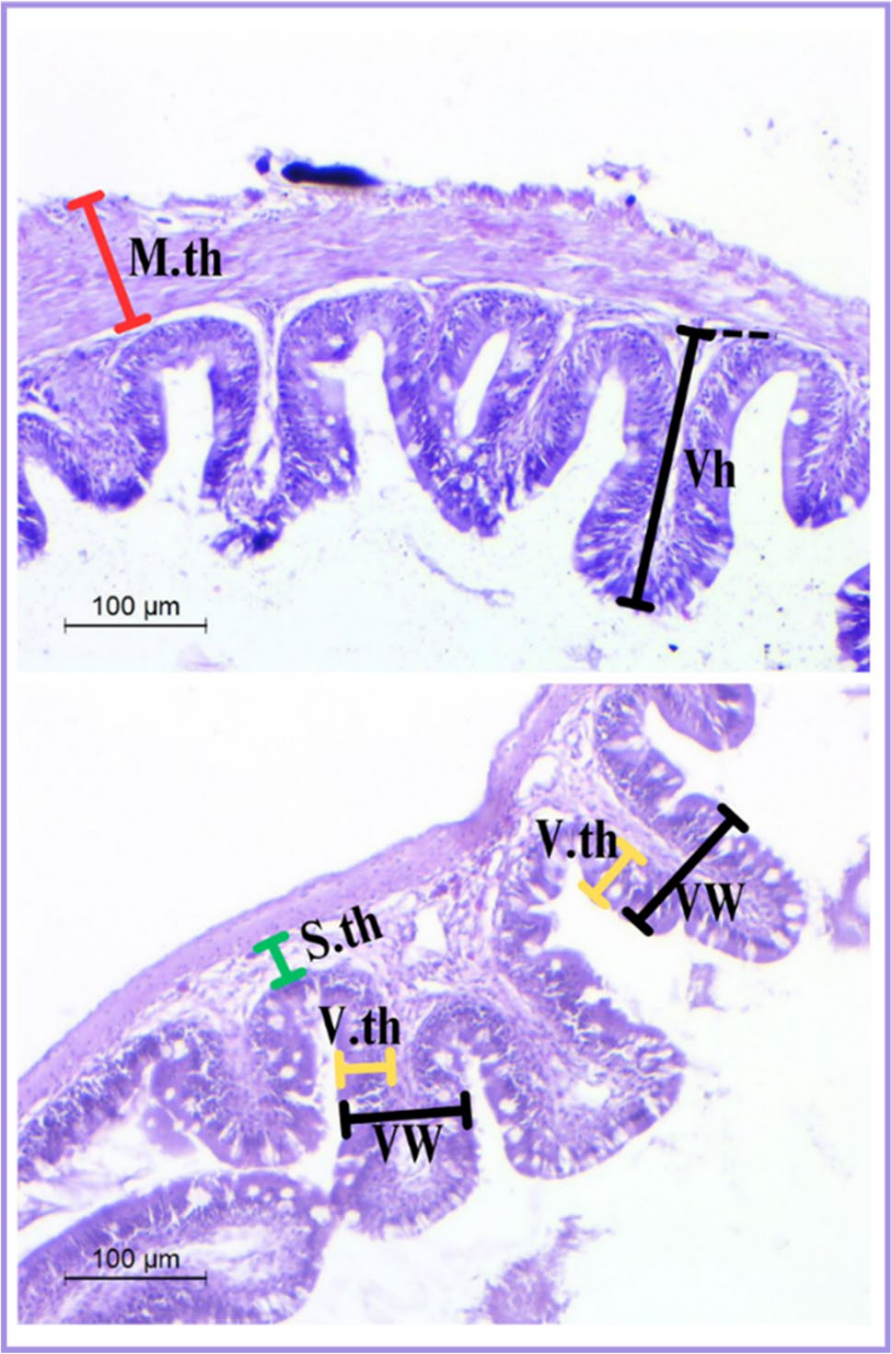
Schematic representation of the intestinal tissue stained with H&E is showing the morphometrical measures, structures, Representative samples illustrated by some symbols: muscularis thickness (M.th); submucosa thickness (S.th); villi height (Vh); villi width (Vw); and villi thickness (V.th)

The control group, according to light microscopy data, is a comparatively undifferentiated muscular tube bordered with normal villi made up of simple columnar epithelium scattered with goblet cells. Notably, these columnar epithelia showed some irregularity Fig. 1. Furthermore, the villi were stubby and leaf-like, which may influence their ability to absorb. Other treated groups had normal layer structures beginning with serosa, muscularis, submucosa, and mucosa. Furthermore, all these groups produced more significant results in tissue architecture, notably in villi histology, than the control group Fig. 2.

#### Quantitative histometric analysis of intestine

The statistical results for the linear measurements and cell density of different experimental groups are shown in Table 3. A significant increase in muscularis thickness was observed in the T_3_ group (27.5 ± 0.76 µm; *P* = 0.007) compared to the other treatments. Mucosal layer measurements revealed that fish in the T_2_ group exhibited the greatest villi height (106.10 ± 1.00 µm; *P* < 0.001) and width (33.35 ± 0.02 µm; *P* = 0.020), while differences in villi thickness were not significant (*P* = 0.299). Additionally, T_2_ recorded the highest lymphocyte count (59.50 ± 0.86; *P* < 0.001) and a markedly greater number of goblet cells (27.75 ± 2.60; *P* = 0.009), indicating enhanced intestinal immune activity. No significant variation was noted in submucosa thickness among the groups (*P* = 0.780). Overall, dietary supplementation with *Bacillus rugosus* NM007, particularly at 0.2 g/kg of feed (T_2_), markedly improved the intestinal histoarchitecture and mucosal immunity of Nile tilapia compared to the control.

**Table 3:** Intestinal histomorphometry of Nile tilapia fed experimental diets supplemented with different doses of *B. rugosus* NM007 for 90 days compared to the control group

	C	T_1_	T_2_	T_3_	*P-value*
Muscularis thickness	9.46 ± 3.65^b^	15.16 ± 0.61^b^	18.80 ± 3.46^ab^	27.5±0.76^a^	0.007
Submucosa thickness	8.60 ± 2.60	6.1 ± 0.81	7.4 ± 1.50	7.6 ± 1.01	0.780
Mucosa layer analysis					
Villi height	43.15 ± 1.99^c^	97.45±1.93^a^	106.10±1.00^a^	85.00 ± 2.88^b^	< 0.001
Villi width	24.46 ± 2.76^b^	25.76 ± 1.67^b^	33.35±0.02^a^	27.05±0.25^ab^	0.020
Villi thickness	10.00±1.05	11.43 ± 1.00	13.35 ± 1.52	10.03 ± 1.55	0.299
Lymphocyte number	15.33 ± 4.33^b^	25.00 ± 2.30^b^	59.50 ± 0.86^a^	22.33 ± 1.4^b^	< 0.001
Goblet cells number	14.33 ± 2.03^b^	16.67 ± 2.02 ^ab^	27.75 ± 2.60^a^	27.67 ± 3.06^a^	0.009

For means in the same row, those with no common superscripts are significantly different at *P* < 0.05. *C* Control, *T*_1_ Treatment 1, (0.1 g/kg of feed), *T*_2_ Treatment 2, (0.2 g/kg of feed), *T*_3_ Treatment 3, (0.3 g/kg of feed)

#### Liver histomorphology

**Fig. 3:**
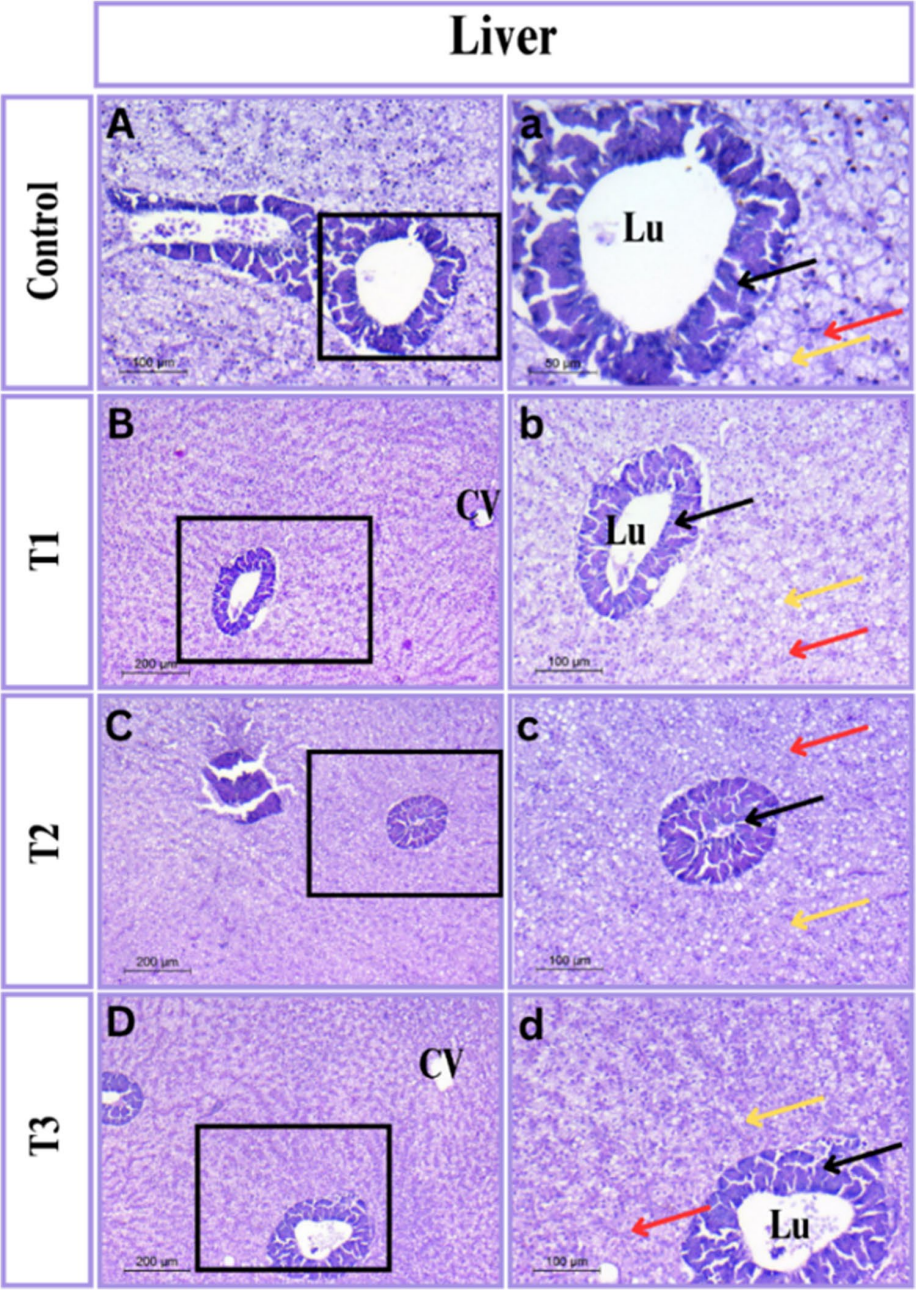
Photomicrograph of liver tissue in Tilapia stained with H&E stain. The right panels (magnification X 200) are higher magnification of the insets of the left panels (magnification X 100). (**A**-**a**) control group; (**B**-**b**) T_1_ group; (**C**–**c**) T_2_ group and (**D**-**d**) T_3_ group. Representative samples illustrated by some symbols: blood sinusoids (red arrow); lumen (Lu); central vein (CV), pancreatic acini (black arrow); and hepatocytes (yellow arrow)

The findings indicated that the tilapia liver histology was normal and that there were no indications of probiotic bacteria-induced toxicity. Its hepatocytes were polygonal with a large, spherical nucleus. The exocrine pancreatic acini exhibited a well-organized cytoarchitecture with a broad lumen in between the liver tissues. Hepatic sinusoids were found to be organized into hepatic cell cords and seemed to be crowded by erythrocytes Fig. 3.

### Hematological analysis

The hematological analysis of Nile tilapia at the end of the 90-day experimental treatment is shown in Table 4. Significant differences (*P* < 0.05) were observed among the treatments for all measured parameters except heterophil percentage (*P* = 0.244). Fish in the T_2_ group (0.2 g/kg of feed) exhibited the highest hemoglobin concentration (14.15 ± 0.43 g/dL; *P* < 0.001), red blood cell count (2.50 ± 0.086 10^6^/mm^3^; *P* = 0.001), and white blood cell count (88.00 ± 4.61 × 10^3^/mm^3^; *P* < 0.001), indicating enhanced oxygen transport and immune response. The T_1_ group showed the highest lymphocyte percentage (%) (83.00 ± 1.73; *P* = 0.043) compared to the control, suggesting stimulation of cellular immunity. Overall, dietary inclusion of *B. rugosus* NM007, particularly at T_2_, positively influenced the hematological parameters of Nile tilapia, reflecting improved physiological and immunological status.

**Table 4:** Hematological parameters of Nile tilapia fed experimental diets supplemented with *B. rugosus* NM007 for 90 days compared to the control group

	C	T_1_	T_2_	T_3_	*P-value*
Hb (g/dl)	10.85 ± 0.14^b^	11.65 ± 0.086^b^	14.15 ± 0.43^a^	13.70 ± 0.057^a^	< 0.001
RBCs (× 10^6^/mm^3^)	1.85 ± 0.086^c^	2.05 ± 0.028^bc^	2.5 ± 0.086^a^	2.25 ± 0.086^ab^	0.001
WBCs (× 10^3^/mm^3^)	52.00 ± 00^b^	55.00 ± 2.59^b^	88.00 ± 4.61^a^	57.50 ± 1.44^b^	< 0.001
Lymphocytes %	78.50 ± 0.28^ab^	83.00 ± 1.73^a^	80.50 ± 2^ab^	68.50 ± 5.48^b^	0.043
Heterophil %	13.00 ± 1.15	8.00 ± 1.15	10.50 ± 0.86	18.50 ± 6.60	0.244

For means in the same row, those with no common superscripts are significantly different at *P* < 0.05. Abbreviations: *Hb* Hemoglobin; *WBCs* White blood cells; *RBCs* Red blood cells. *C* Control, *T*_1_ Treatment 1, (0.1 g/kg of feed), *T*_2_ Treatment 2, (0.2 g/kg of feed), *T*_3_ Treatment 3, (0.3 g/kg of feed)

### Biochemical parameters

The results of the biochemical analysis of Nile tilapia after 90 days of experimental diets is displayed in Table 5. Statistical analysis revealed significant differences (*P* < 0.05) among treatments for all parameters except globulins (*P* = 0.087), urea (*P* = 0.241), and creatinine (*P* = 0.459). Fish in the T_3_ group (0.3 g/kg of feed) exhibited the highest total protein (4.25 ± 0.20 g/dL; *P* = 0.004) and albumin (1.35 ± 0.02 g/dL; *P* = 0.013) levels, indicating improved protein synthesis and liver function. Conversely, the lowest alanine aminotransferase (ALT) and aspartate aminotransferase (AST) activities were recorded in the T_2_ group (6.50 ± 0.28 U/L and 41.00 ± 3.46 U/L, respectively; *P* < 0.05), suggesting reduced hepatic stress and better liver integrity. Cholesterol levels were highest in T_1_ (183.00 ± 1.44 mg/dL; *P* < 0.001). Alkaline phosphatase (ALP) and bilirubin also varied significantly among treatments, with the highest levels recorded in the T_3_ group (152.00 ± 5.10 U/L; *P* = 0.015 and 0.085 ± 0.002 mg/dL; *P* = 0.003, respectively). Glucose levels decreased markedly in T_2_ (21.50 ± 1.44 mg/dL; *P* < 0.001), reflecting improved metabolic efficiency.

**Table 5:** Biochemical parameters of Nile tilapia fed experimental diets supplemented with *B. rugosus* NM007 for 90 days compared to the control group

	C	T_1_	T_2_	T_3_	*P-value*
ALT (U/L)	12.50 ± 0.86^a^	13.00 ± 0.57^a^	6.50 ± 0.28^b^	13.00 ± 0.57^b^	< 0.001
AST (U/L)	80.50 ± 7.21^a^	71.00 ± 2.80^a^	41.00 ± 3.46^b^	80.5 ± 4.33^a^	< 0.001
TP (g/dl)	3.21 ± 0.14^c^	3.30 ± 0.11^bc^	4.00 ± 0.17^ab^	4.25 ± 0.20^a^	0.004
Alb (g/dl)	0.90 ± 0.01^b^	1.25 ± 0.28^a^	1.25 ± 0.14^a^	1.35 ± 0.02^a^	0.013
Globulins (g/dl)	2.31 ± 0.14	2.05 ± 0.14	2.75 ± 0.31	2.90 ± 0.32	0.087
Cholesterol (mg/dl)	131.00 ± 3.46^c^	183.00 ± 1.44^a^	178.50 ± 1.44^a^	152.00 ± 3.46^b^	< 0.001
Alk.ph (U/L)	133.00 ± 2.30^ab^	124.00 ± 1.73^b^	144.00 ± 7.50^ab^	152.00 ± 5.10^a^	0.015
Bili (mg/dL)	0.03 ± 0.01c	0.05 ± 0.005^bc^	0.06 ± 0.002^ab^	0.085 ± 0.002^a^	0.003
Urea (mg/dL)	3.00 ± 0.01	4.50 ± 0.86	4.50 ± 0.288	4.00 ± 0.57	0.241
Creatinine (mg/dL)	0.26 ± 0.01	0.32 ± 0.04	0.29 ± 0.03	0.32 ± 0.01	0.459
Glucose (mg/dL)	32.00 ± 0.57^a^	32.50 ± 2^a^	21.50 ± 1.44^b^	24.50 ± 0.86^b^	< 0.001

For means in the same row, those with no common superscripts are significantly different at *P* < 0.05

Abbreviations: *ALT* Alanine Aminotransferase; *AST* Aspartate Aminotransferase; *TP* Total Protein; *Alb* Albumin, *Alk.ph* Alkaline phosphatase, *Bili* Bilirubin

*C* Control, *T*_1_ Treatment 1, (0.1 g/kg of feed), *T*_2_ Treatment 2, (0.2 g/kg of feed), *T*_3_ Treatment 3, (0.3 g/kg of feed)

These results suggest that supplementation with *Bacillus rugosus* NM007, particularly at T_2_ and T_3_, improves liver function and metabolic health in Nile tilapia while reducing biochemical indicators of hepatic stress.

### Antioxidant capacity

**Fig. 4:**
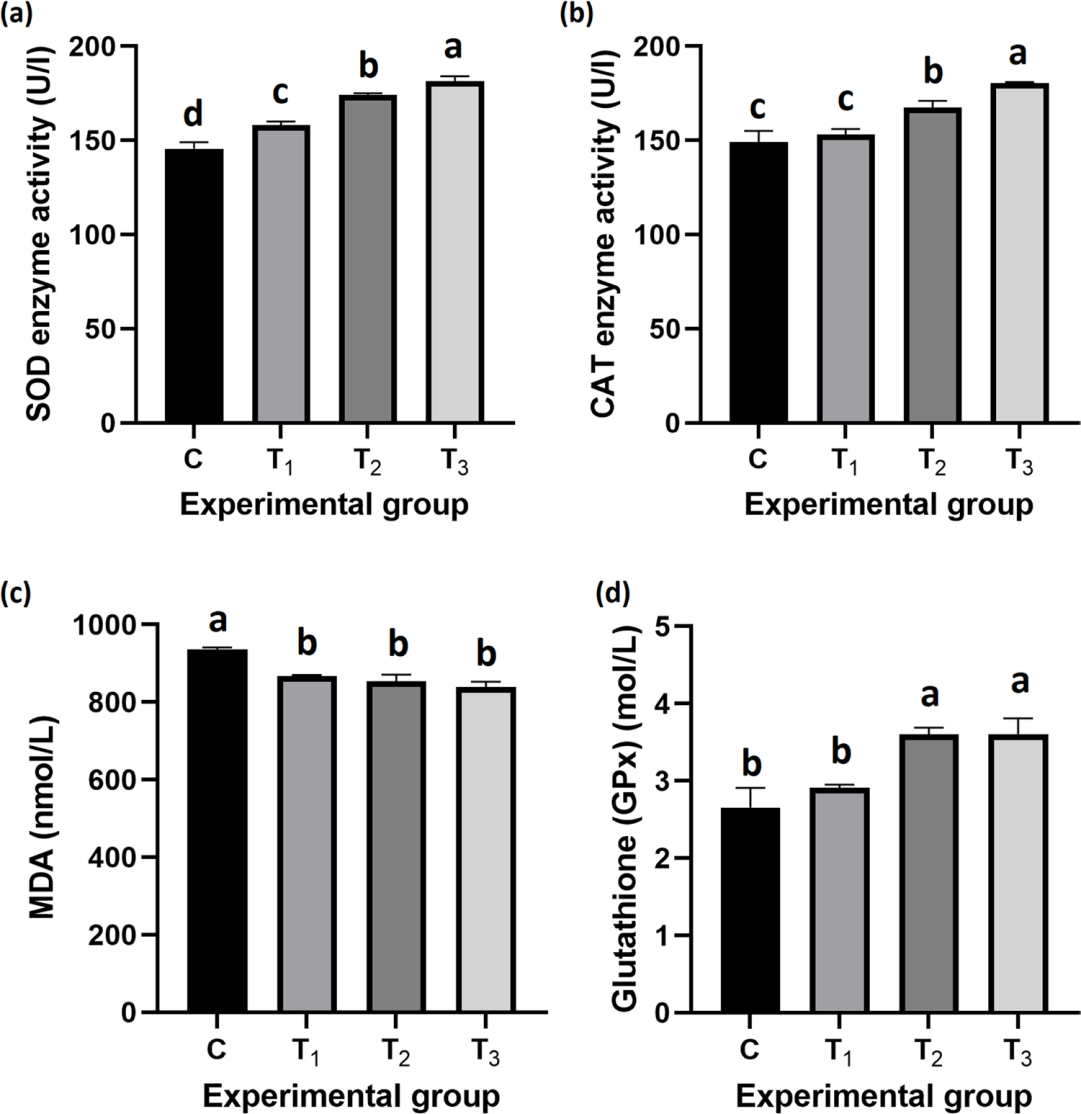
Impact of dietary *B. rugosus* NM007 on fish oxidative stress markers. This figure presents the mean (± SEM) activities of key oxidative stress parameters in fish following 90 days of feeding on diets supplemented with *B. rugosus* NM007. It shows: (**a**) Superoxide Dismutase (SOD) activity (U/L); (**b**) Catalase (CAT) activity (U/L); (**c**) Malondialdehyde (MDA) activity (nmol/L); and (**d**) Glutathione (GPx) activity (mol/L). The columns (mean ± SEM) with different letters indicate significant differences (*P* < 0.001, one-way ANOVA)

Figure 4 shows that the activities of antioxidant enzymes significantly increased in all probiotic-treated groups compared to the control. Superoxide dismutase (SOD) activity was highest in T_3_ (181.50 ± 1.44 U/mL; *P* < *0.001*), catalase (CAT) activity was highest in T_3_ (180.50 ± 0.60 U/L; *P* < *0.001*), and glutathione peroxidase (GPx) activity was highest in T_2_ (3.63 ± 0.075 nmol/L; *P* < *0.001*). In contrast, malondialdehyde (MDA) levels, an indicator of lipid peroxidation, were highest in the control group (936.50 ± 0.33 nmol/L; *P* < *0.001*) and decreased significantly in the treated groups, reaching the lowest value in T_3_ (839.50 ± 7.74 nmol/L; *P* < *0.001*).

These results suggest that dietary supplementation with *Bacillus rugosus* NM007 enhances antioxidant defense in Nile tilapia while reducing oxidative stress.

### Digestive enzymes

#### Digestive enzymes activity in serum

**Fig. 5:**
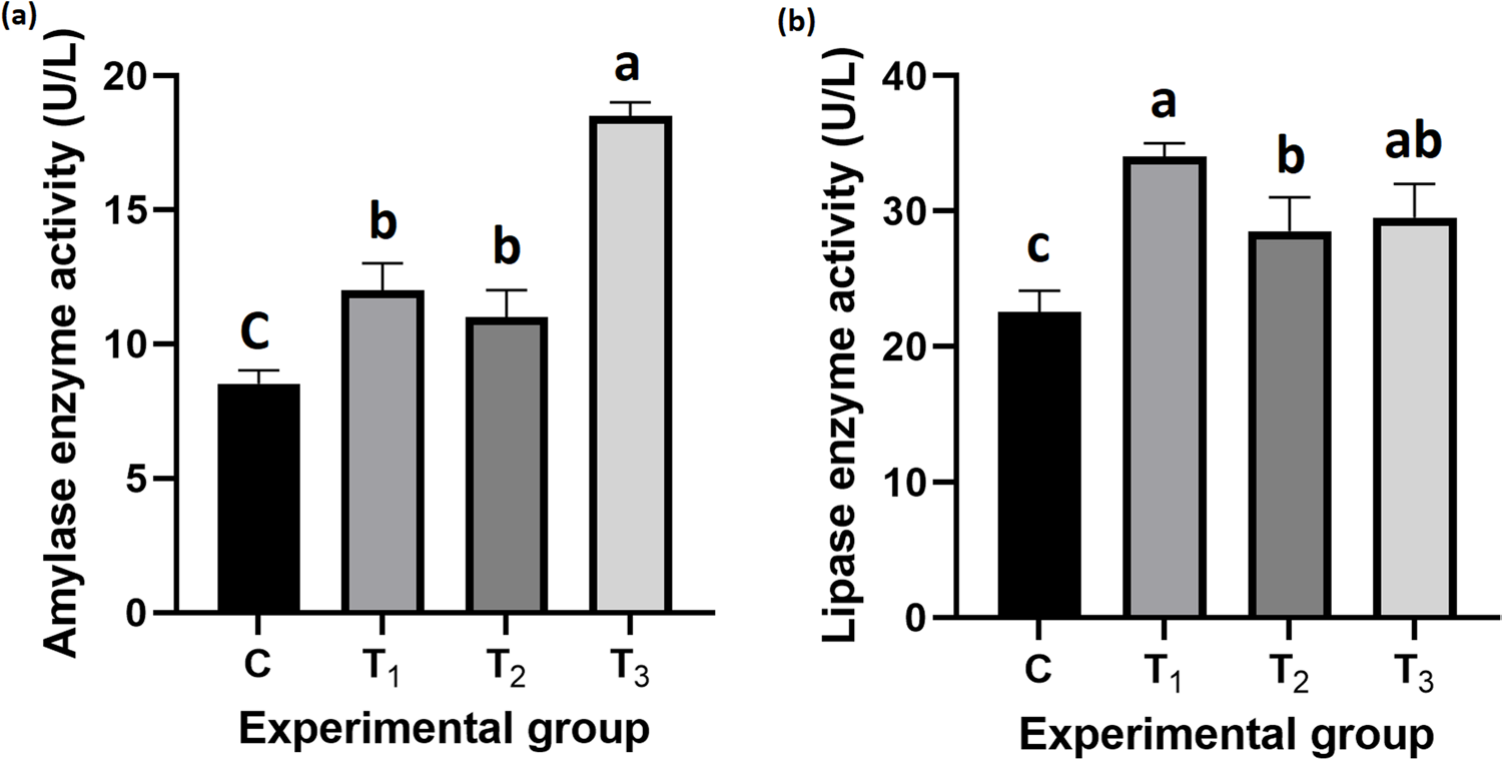
Effect of dietary *B. rugosus* NM007 supplementation on serum digestive enzyme activity in Nile tilapia. The figure compares the mean (± SEM) activities of two key serum digestive enzymes, amylase (U/L) (**a**) and lipase (U/L) (**b**), in Nile tilapia fed a *B. rugosus* NM007-supplemented diet versus a control diet over 90 days. The columns (mean ± SEM) with different letters indicate significant differences (*P* < 0.001, one-way ANOVA)

Figure 5 shows the activity of digestive enzymes in fish serum. Amylase activity was significantly higher in all probiotic-treated groups compared to the control, with the highest value observed in T_3_ (18.50 ± 0.28 U/L; *P* < *0.001*). Similarly, lipase activity significantly increased in the treated groups, with the highest activity recorded in T_1_ (34.00 ± 0.57 U/L; *P* < *0.001*).

#### Digestive enzymes activity in intestine

**Fig. 6:**
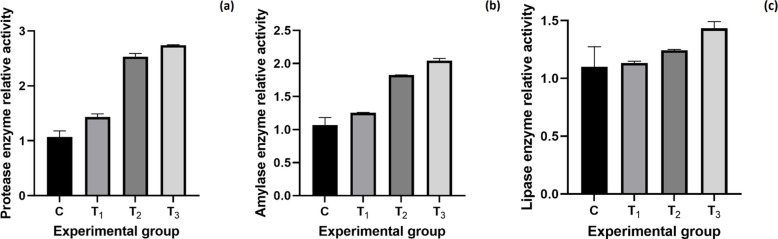
Modulation of intestinal digestive enzymes by dietary *B. rugosus* NM007.This figure shows the relative activity (mean ± SEM) of three key intestinal enzymes, protease (**a**), amylase (**b**), and lipase (**c**) in Nile tilapia fed a *B. rugosus* NM007-supplemented diet for 90 days versus a control diet

Figure 6 shows the relative activity of intestinal digestive enzymes in Nile tilapia. Protease activity was higher in all probiotic-treated groups compared to the control, with the highest value observed in T_3_ (2.74 ± 0.005). Amylase activity was also elevated in the treated groups, reaching the highest level in T_3_ (2.04 ± 0.02). Similarly, lipase activity increased in the probiotic-supplemented groups, with the highest value recorded in T_3_ (1.43 ± 0.04).

### Immune response

**Fig. 7:**
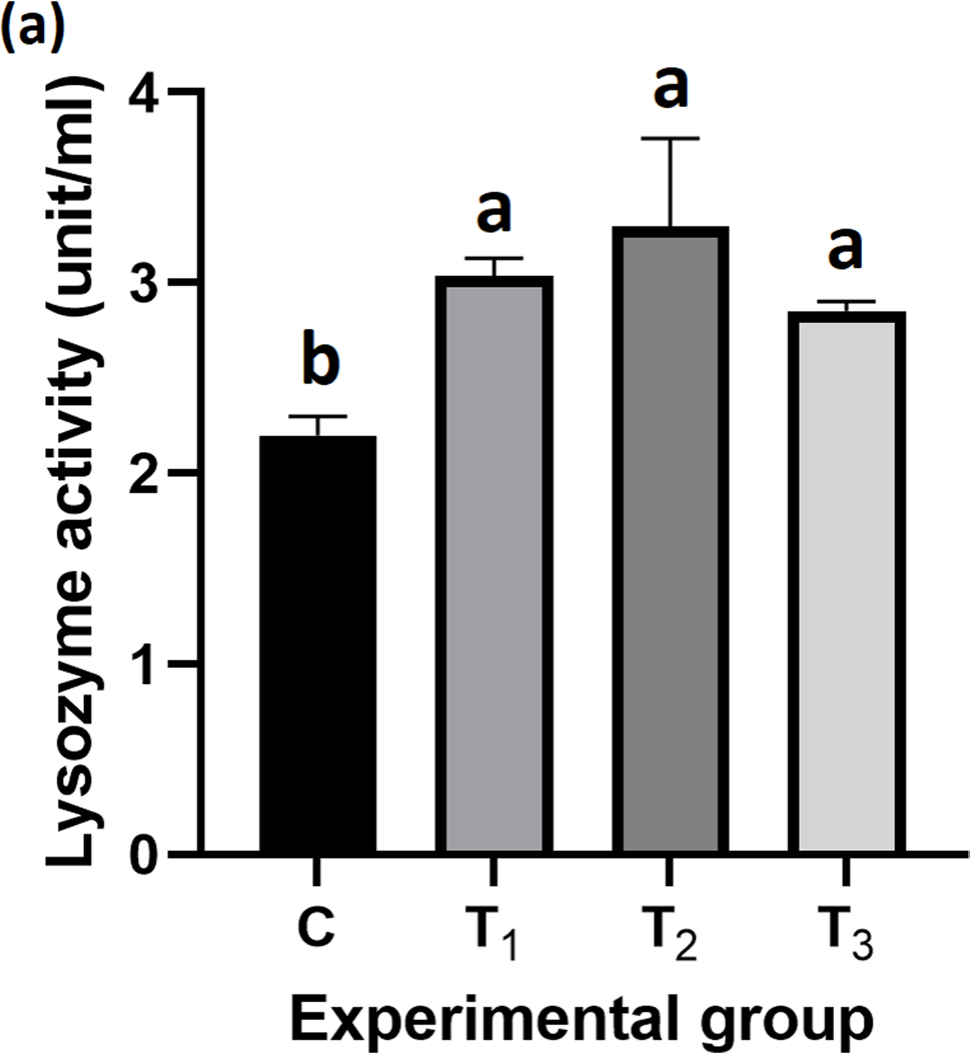
Lysozyme activity (Unit/mL) in Nile tilapia serum. Lysozyme activity (mean ± SEM) in Nile tilapia following 90 days of feeding with *B. rugosus* NM007 supplementation. The columns (mean ± SEM) with different letters indicate significant differences (*P* = 0.003, one-way ANOVA)

Figure 7 shows the lysozyme activity, an indicator of innate immune response, in Nile tilapia. Lysozyme activity was significantly higher in all probiotic-supplemented groups compared to the control, with the highest level observed in T_2_ (3.30 ± 0.26 ng/mL; *P* = *0.003*).

### Whole body composition

The percentages of protein, fat, ash, carbohydrates, moisture, and dry matter in the whole fish body show a significant difference (*P* < *0.05*) between all experimental groups and the control group (Table 6). The highest protein percentage was reported in T_2_, and the highest percentage of fat was reported in T_3_.

**Table 6:** Proximate body composition (mean ± SEM) of Nile tilapia fed diets supplemented with *B. rugosus* NM007 for 90 days compared to the control group

	C	T_1_	T_2_	T_3_	*P-value*
Protein %	15.5 ± 0.01^b^	15.64 ± 0.02^a^	15.72 ± 0.02^a^	15.43 ± 0.01^b^	< 0.001
Fat %	4.58 ± 0.05^c^	4.65 ± 0.01^b^	4.65 ± 0.05^b^	4.72 ± 0.08^a^	< 0.001
Ash %	3.56 ± 0.02^b^	3.54 ± 0.01^b^	3.73 ± 0.01^a^	3.52 ± 0.03^b^	< 0.001
Carbohydrate %	2.06 ± 0.02^a^	1.75 ± 0.06^c^	1.15 ± 0.08^d^	1.97 ± 0.08^b^	< 0.001
Moisture %	74.30 ± 0.01^c^	74.42 ± 0.01^b^	74.75 ± 0.01^a^	74.36 ± 0.04^bc^	< 0.001
Dry matter %	25.70 ± 0.01^a^	25.58 ± 0.07^b^	25.25 ± 0.04^c^	25.64 ± 0.04^ab^	< 0.001

For means in the same row, those with no common superscripts are significantly different at *P* < 0.05. *C* Control, *T*_1_ Treatment 1, (0.1 g/kg of feed), *T*_2_ Treatment 2, (0.2 g/kg of feed), *T*_3_ Treatment 3, (0.3 g/kg of feed)

## Discussion

The growth rates and effectiveness of feed consumption were determined by using *Bacillus* spp. supplemented diets significantly increased, suggesting that *Bacillus* spp. probiotics may reduce stressors and promote fish welfare. This has been noted in tilapia (Elsabagh et al. 2018; Moustafa et al. 2021; Mugwanya et al. 2022; Ghalwash et al. 2022), and also other fish species (Mandiki et al. 2011; Gupta et al. 2016; Kewcharoen and Srisapoome 2019; Yang et al. 2020). Numerous research has revealed that *Bacillus* spp. can colonize the fish gut, produce digestive enzymes, and detoxify harmful feed components, which collectively maintain a healthy gut and increase the uptake, utilization and digestibility of nutrients. Recent studies also emphasize that *Bacillus* probiotics can modulate gut microbiota composition, leading to improved gut barrier function and pathogen exclusion, which aligns with our observed intestinal histomorphometry improvements (Li et al. 2021; Sun et al. 2023, 2025; Tang et al. 2024).

Moreover, improving feed intake, feed efficiency, and growth rates are becoming a trend in aquaculture practices (El-Kassas et al. 2020; Abozeid et al. 2021; Elkaradawy et al. 2021). In both freshwater and marine water fish, the utilization of probiotics in the form of a single strain or multiple strains has many beneficial effects on improving blood parameters, immunological response, feed utilization, and growth performance (Bidhan et al. 2014; Al-Deriny et al. 2020; Khalafalla et al. 2020).

The findings of this study demonstrated that the effects of *Bacillus rugosus* NM007 probiotics inclusion in diet enhanced the growth parameters, feed conversion rate, protein efficiency and intestinal weight of the Nile tilapia as compared to the control group. The most significant differences were recorded in the T_2_ group in which fish fed diets supplemented with *Bacillus rugosus* NM007 potential probiotic at 0.2 g/kg of feed; additionally, the intestinal weight also showed the best result in group T_2_. Silva et al. (2021) similarly, they observed that *Bacillus* strains improved feed conversion ratio (FCR) and weight gain after 90 days, which supports our findings. The enhanced growth might be directly linked to the increase in intestinal digestive enzyme activity (protease, amylase, and lipase) observed in the treated groups, suggesting a functional mechanism beyond just observing weight gain. Notably, while feed intake (FI) and hepato-somatic Index (HSI) did not change significantly, the observed improvements in growth and FCR indicate that the probiotic enhances nutrient utilization efficiency rather than simply increasing appetite, highlighting its role in optimizing feed conversion. Similarly, studies have shown that Nile tilapia fed diets supplemented with *Bacillus* probiotics exhibit improved growth performance and feed conversion ratio (FCR) (Elsabagh et al. 2018; Abdel-Ghany et al. 2020). Probiotics also can use available nutrients, whereas for the host, they can synthesize useful nutrients like vitamins, amino acids, and fatty acids, and also help in the improvement of intestinal health by reducing the effects of pathogenic microorganisms (Zaineldin et al. 2021). In addition, several studies have supported the effect of probiotic use, in particular of *Bacillus* spp. It can produce several enzymes such as proteases, amylase and lipase to enhance the growth and morphometric parameters of the fish intestine (Standen et al. 2015; Elsabagh et al. 2018; El-Kassas et al. 2020; Zaineldin et al. 2021; Ghalwash et al. 2022). In the current study, there was intestinal histo-morphological improvement in the absorptive surface epithelium of the tilapia gut fed a feed supplemented with *B. rugosus* NM007 potential probiotic. Specifically, the absorptive surface area manifested as increased intestinal villi height, width, and thickness in the T_2_ group fed *Bacillus rugosus.* These observations may be due to the fact that probiotics have a direct impact on gut microbiota, which, in turn, promotes the production of carbohydrates in the fish gut, resulting in the generation of short-chain fatty acids, propionic, butyric, and acetic acid, which has a stimulatory effect on epithelial proliferation at higher enrichments (Falcinelli et al. 2016; Venegas et al. 2019).

For blood parameter analysis, the fish fed on a diet supplemented with *Bacillus* probiotics revealed an enhanced hematological profile, including hemoglobin, RBCs and WBCs counts than the control group (Tabassum et al. 2021). Furthermore, numerous studies have shown an increase in the hematological parameters of Nile tilapia fed with *Bacillus*-based probiotics (Liu et al. 2021; Ghalwash et al. 2022). According to Sayed Hassani et al. (2020), hemoglobin levels in Nile tilapia fed a diet supplemented with *Bacillus* species significantly improved. In this study, there are significant differences between the groups that fed diet supplemented with *B. rugosus* NM007 and the control group, especially the T_2_ group, which had the most significant improvement in hemoglobin levels. This considerable increase is suggesting better oxygen delivery to the tissues and cells, hence increasing the aerobic capacity of cells and energy utilization (Islam et al. 2022). High hemoglobin concentrations can also increase the ability to continue with activities due to more oxygen-carrying capacity, which is useful for organisms in stressed or active habitats (Islam et al. 2022). The value pertaining to the T_2_ treatment also turned out to be the highest for the RBC count, reflecting the increase in erythropoiesis. An increase in RBCs helps in the delivery of oxygen to all tissues, metabolism, and development of tissues and organs in the body. Moreover the highest WBCs was observed in T_2_ that reflects good immunity (Islam et al. 2022). Increased WBCs count is favorable as an indicator, since it represents a better response to the immune system and general resistance to diseases (Islam et al. 2022). Moreover, the results suggest that *B. rugosus* NM007 plays a role in increasing the hemoglobin, RBCs, and WBCs and other hematological parameters when compared with other studies using different Lactobacilli and *Bacillus* spp. (Elsabagh et al. 2018; Ghalwash et al. 2022). This suggests that *B. rugosus* NM007 might be effective at enhancing oxygen-carrying capacity and overall blood health, potentially improving the health of the fish.

The highest value of the lymphocyte percentage was given by the T_1_ treatment corresponded to powerful adaptive immunity. Lymphocytes are central to the body’s immune system, as they are involved in the production of antibodies and control of immune responses (Yu et al. 2020). The increase in the percentage of lymphocytes implies a better capability for countering infections as well as boosting surveillance of immune cells (Farag et al. 2021; Mazziotta et al. 2023; Mathew et al. 2025). The significantly higher goblet cell number observed in the T_2_ group suggests that dietary supplementation with *Bacillus rugosus* NM007 enhances intestinal immune function in Nile tilapia. Goblet cells are critical for mucus secretion, which forms a protective barrier against pathogens (Cabillon and Lazado 2019; Gustafsson and Johansson 2022). These findings indicate that probiotics not only support the structural integrity of the intestinal mucosa but also stimulate local immune defenses, potentially improving disease resistance.

Furthermore, the findings of this study have potential industrial and economic implications. Incorporating *B. rugosus* NM007 into commercial tilapia diets could enhance growth performance, feed efficiency, and overall fish health, potentially reduce production costs and improve profitability in aquaculture operations.

A decrease in AST levels was observed in fish groups that were fed a diet supplemented with *B. rugosus* NM007 potential probiotic, and it is interesting to note that the T_2_ group showed significantly lower ALT and AST levels, suggesting hepatoprotective and enhanced metabolic activity effects of *B. rugosus* NM007 (Huang et al. 2021; Mozanzadeh et al. 2021; Wu et al. 2022), and this is also supported by several studies that have reported the beneficial effects of *Bacillus* spp. probiotics in shrimp (Ziaei-Nejad et al. 2006; Liu et al. 2009). The same was also reported in Nile tilapia received a diet supplemented with *Bacillus* spp. (Liu et al. 2009). In this regard, the results indicate the possibility of using *Bacillus*-based probiotics as an effective liver protector, since the rise or fall of AST levels is seen as an indication that there is injury occurring within the liver cells, such as hepatocyte necrosis (Wang et al. 2023). Moreover, T_3_ treatment had the highest value of alkaline phosphatase activity. Alkaline phosphatase is an enzyme that is involved in bone mineralization and the liver and its upregulation may indicate enhanced bone metabolism and liver function (Bozorgzadeh et al. 2023; Zafar and Khan 2024). This finding suggests that there is a positive effect of *B. rugosus* NM007 potential probiotic on bone development and liver function.

Since stress and oxidative stress can have an impact on fish's antioxidant status, the activities of antioxidant enzymes are also used to assess it (Feng and Wang 2020; Abdel-Latif et al. 2023). Compared to the control group in this study, the activities of antioxidant enzymes in fish fed *B. rugosus* NM007 potential probiotic-related diets were higher. Consequently, the average superoxide dismutase (SOD) was significantly higher in treated groups, especially the T_3_ groups. Similar to the results observed with SOD, CAT activity was significantly increased in the T_3_ group, which is higher than the value in the control group. Furthermore, the GPx content of fish groups fed with probiotic-supplemented diets was higher than the control group in all treatment groups. The GPx levels for T_2_ and T_3_ groups were raised significantly more than the control group and T_3_ had the highest value. *B. rugosus* NM007 has been found useful in increasing the activity of antioxidant enzymes in fish’s blood. This increase in the activity of SOD, CAT and (GPx) in treated groups is supported by several previous studies (Abdella et al. 2020; Hoseinifar et al. 2020; Bañuelos-Vargas et al. 2021; Mohammadi et al. 2021; Naiel et al. 2022). These studies suggested that the application of probiotics in various fish species and in various rearing environments makes use of supplementation beneficial in enhancing the wellbeing of fish as well as their resistance to oxidative stress. Further, our study noted the following: all treated groups recorded significantly lower levels of MDA than the control group, especially T_3_ group. These results suggest the MDA levels depict less lipid peroxidation and better cell defense. Low concentrations of MDA appear to be indicative of decreased oxidative stress byproducts that impact cell membranes in a negative manner, thus maintaining the structural and functional integrity of cells (Ghalwash et al. 2022).

The overall improvement in non-specific immune performance was recorded in fish fed diet supplemented with *B. rugosus* NM007 potential probiotic, which showed a significant difference in lysozyme activity than that of the control group, especially T_2_ group. These findings show the benefits of *B. rugosus* NM007 potential probiotic to enhance the immunity of the fish. This is supported by several studies that recorded an increase in the immunological state of Nile tilapia while using probiotics over the control group (Hasan and Banerjee 2020; Kazuń et al. 2020; Putra et al. 2021; Ghalwash et al. 2022). Studies have shown that the immune response in young tilapia fish can be improved by incorporating probiotic bacteria such as *Bacillus* strains into their feed (Harpeni et al. 2018; Olmos et al. 2020; Ringø et al. 2020; Elsegeny et al. 2025). Another study showed higher levels of lysozyme in tilapia fed with *Bacillus* strains as a probiotic (Demers and Bayne 1997; Mandiki et al. 2011; Kim et al. 2017; Amoah et al. 2019; Sayed Hassani et al. 2020; El-Saadony et al. 2021; Ghalwash et al. 2022). Furthermore, several studies have demonstrated significant improvements in lysozyme activity in fish receiving probiotics compared to those that not receiving them (Kim et al. 2017). The immune-boosting effect has been demonstrated to be caused by the release of antimicrobial substances by bacteria like *Bacillus* that can combat harmful microorganisms in fish (Mandiki et al. 2011; Selim and Reda 2015; Amoah et al. 2019; Pronina et al. 2021).

In the whole body composition analysis of fish that fed a diet supplemented with *B. rugosus* NM007, there were statistical differences between the treatments and the control, with the highest protein and fat percentage being observed in T_2_ and T_3,_ respectively. These results may be attributed to improved fish performance in response to probiotic applications. Similar results were observed by (Khalafalla et al. 2020).

Aquatic animals' growth depends on the digestive tract's ability to break down, absorb, and metabolize different nutrients—processes aided by gut-based digestive enzymes (Ziaei-Nejad et al. 2006; Dawood et al. 2019). Aquatic animals' capacity to digest food is indicated by the activity of these enzymes, which can then be linked to the animals' growth performance. As the application of probiotics for boosting growth performance has been rising in aquaculture, the interest in the digestive enzyme activity in the intestines of aquaculture species has been growing (Meng et al. 2010; Méndez-Martínez et al. 2018; Zuo et al. 2019). In the present investigation, protease, amylase, and lipase values were raised significantly in the intestine of fish in *B. rugosus* NM007 probiotic associated groups in both the blood and intestine of the fish. This increase could be ascribed to improved secretion of digestive enzymes connected to probiotic administration. These results imply that nutrient digestion and absorption were more effective in the treated groups, suggesting the potential benefits of probiotics in aquaculture.

Despite the promising results, this study has some limitations. The first limitation is that it was under lab-controlled conditions. A 90-day trial may not accurately reflect the long-term effects of probiotics on fish health and growth. Thus, the expansion of the study to field conditions is important for result validation in the grow-out period. The long-term persistence of the observed benefits, such as elevated antioxidant levels and improved liver function, after discontinuing probiotic supplementation remains uncertain. Future research should explore these long-term effects, optimal dosing strategies, and the mechanisms underlying gut microbiota modulation, as well as evaluate its efficacy across different fish species and farming conditions to support the practical implementation in commercial aquaculture.

## Conclusion

The results of this study demonstrate that dietary supplementation of Nile tilapia with *Bacillus rugosus* NM007 at 0.2 g/kg of feed enhances growth performance and nutrient utilization, in addition to intestinal and liver health. It showed improvements in the hemato-biochemical and immune-oxidative responses. These findings support the potential application of *B. rugosus* NM007 to promote fish growth, optimize feed efficiency, and strengthen the immunity of the fish. Future research should explore long-term effects, optimal dosing strategies, and the mechanisms underlying gut microbiota modulation, as well as evaluate its efficacy across different fish species and real farming conditions to support the practical implementation in commercial aquaculture.

## Data Availability

The data included in this study can be accessed by contacting the corresponding author.
